# Numerical Investigation of Graphene as a Back Surface Field Layer on the Performance of Cadmium Telluride Solar Cell

**DOI:** 10.3390/molecules26113275

**Published:** 2021-05-28

**Authors:** Devendra KC, Deb Kumar Shah, M. Shaheer Akhtar, Mira Park, Chong Yeal Kim, O-Bong Yang, Bishweshwar Pant

**Affiliations:** 1 Electrical Department, Gabriel Elektro AS, 9700 Lakselv, Norway; devendrakc25@gmail.com; 2 School of Semiconductor and Chemical Engineering, Jeonbuk National University, Jeonju 54896, Korea; dkshah149@gmail.com; 3 Graduate School of Integrated Energy-AI, Jeonbuk National University, Jeonju 54896, Korea; shaheerakhtar@jbnu.ac.kr; 4 New and Renewable Energy Materials Development Center (NewREC), Jeonbuk National University, Jeonbuk 56332, Korea; kimbo@jbnu.ac.kr; 5 Carbon Composite Energy Nanomaterials Research Center, Woosuk University, Wanju, Chonbuk 55338, Korea; wonderfulmira@woosuk.ac.kr

**Keywords:** CdTe solar cell, graphene, back surface, efficiency, simulation

## Abstract

This paper numerically explores the possibility of ultrathin layering and high efficiency of graphene as a back surface field (BSF) based on a CdTe solar cell by Personal computer one-dimensional (PC1D) simulation. CdTe solar cells have been characterized and studied by varying the carrier lifetime, doping concentration, thickness, and bandgap of the graphene layer. With simulation results, the highest short-circuit current (I_sc_ = 2.09 A), power conversion efficiency (η = 15%), and quantum efficiency (QE~85%) were achieved at a carrier lifetime of 1 × 10^3^ μs and a doping concentration of 1 × 10^17^ cm^−3^ of graphene as a BSF layer-based CdTe solar cell. The thickness of the graphene BSF layer (1 μm) was proven the ultrathin, optimal, and obtainable for the fabrication of high-performance CdTe solar cells, confirming the suitability of graphene material as a BSF. This simulation confirmed that a CdTe solar cell with the proposed graphene as the BSF layer might be highly efficient with optimized parameters for fabrication.

## 1. Introduction

Modifications in the physical features of photovoltaic devices lead to improvement in the efficiency of a solar cell [1,2,3]. In CdTe/CdS solar cells, a metal layer with the work function ≥5.7 eV is needed to achieve a low contact resistance for proper functionality and sustainability. Generally, the Schottky barrier of a solar cell is rectified by a highly doped p-CdTe and insertion of a back surface field (BSF) layer at the CdTe/metal layer [4,5]. The BSF is an additional part, which consists of a heavily doped layer next to an absorber layer in the modern CdTe solar cell, i.e., a rear surface of the cell [6]. The highly doped BSF region acts as a barrier layer for minority mobile charge carriers at the CdTe/metal interface. The main purpose of providing a BSF layer in the structure of solar cells is to reduce the barrier width in the valence band and to reduce the recombination losses at the back surface of the CdS/CdTe solar cell [7,8,9]. The main technical problems of CdS/CdTe solar cells are related to higher efficiency, less material usage, and stable back contact formation [10,11]. These problems should be addressed and numerically evaluated to discover the unseen potentiality of CdS/CdTe solar cells for higher cell performance [10,11,12,13]. CdTe has good electronic properties, a high optical absorption coefficient of over 1 × 10^4^/cm, and has a direct energy bandgap of 1.45 eV, which is very near to the optimal bandgap for solar cells [10,14]. However, a lower thickness of the absorber layer is required, which can help to minimize cell material usage and reduce the cost of the manufacture of cells [10,15,16]. Due to the limitation of the reduction in the thickness of the absorber layer, the insertion of a thin back surface field (BSF) is essential to maintain a higher efficiency of CdTe solar cells [10,17]. The BSF layer of solar cells enhances the efficiency and FF because it degrades the shunting effects in the absorber layer [18]. A thickness of 1 μm for a BSF is sufficient for the recombination process of carriers at the interface of layers in the solar cells [7,19]. Providing a BSF layer in a CdTe solar cell may lead to some improvements like a thinner absorber layer with significant performance and reduction in cell fabrication cost [20]. The BSF layer helps the hole collection capability of CdTe cells due to the penetrating network and offers massive electrical transference routes to tie the individual graphene sheets [18,21,22]. Low-cost materials like graphene could be used as ultimate back contact without interrupting cell performance and stability.

The exploration of a stable and efficient back contact layer is important for the long-term stability of CdTe/CdS solar cells. CdTe has a high electron affinity and thus a high work function element is required to generate a good ohmic contact on p-type CdTe [10,23]. Graphene materials exhibit high carrier mobility (2 × 10^5^ cm^2^/V·s) at room temperature, a high work function of 5.5 eV [18,24], excellent transparency (as they absorb light by ~2.3% across most of the ultraviolet (UV) and visible spectrum), marvelous thermal conductivity (~10^3^ W/m·K) [25], and a high melting point (~5000 K) [26]. Besides the atom-layer structure, they have a large surface area and graphene sheets have high flexibility. Therefore, low-cost graphene can be used in a wide range of applications, such as chemical sensors [27,28], medical devices [29], photodetectors [30], energy storage, [31], manufacturing roll-to-roll electronic devices [32], and solar cells [33,34]. Bhandari et al., in 2021, successfully incorporated added FeS_2_-NC back contact in CdTe solar cells and showed good thermal stability under initial tests. Devices prepared with untreated FeS_2_-NC back contacts display a strong “S-kink” behavior which correlates with a high hole-transport barrier arising from inter-NC organic surfactant molecules, but maximum efficiency was only 12.7% [7]. Liang et al., in 2012, successfully combined Cu nanowire-doped graphene (Cu NWs/graphene) as the back contact layer in thin-film CdTe solar cells. The efficiency of solar cells with Cu NWs/graphene was up to 12.1% [18]. The drawback with the Cu-based back contact layer is the Cu diffusion with the grain boundaries as well as across the junction.

In this work, a CdTe solar cell was designed and simulated using the PC1D simulation tool to investigate the influence of carrier lifetime, doping concentration, bandgap, and thickness of the BSF layer on the conversion efficiency of the CdTe solar cell. The carrier lifetime, doping concentration, and thickness of the BSF layer are critical parameters for the PV properties of solar cells. This modeling study aimed to check the effect of carrier lifetime, doping concentration, and thickness of BSF layer on the I_sc_, V_oc_, and efficiency and recommend the best possible combination for fabrication.

## 2. Materials and Methods

### 2.1. Solar Cell Structure and BSF Layer

**Figure 1:**
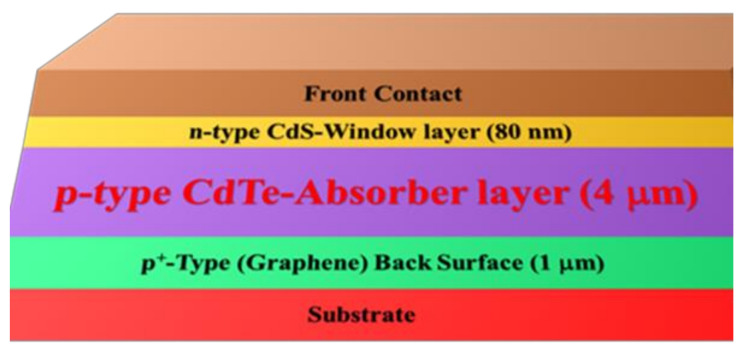
The device structure of graphene back surface-based CdTe solar cell.

The purpose of numerical investigation in the PV cell analysis is to examine the validity of the projected device structure’s arranged cell geometry and cell efficiency. In this proposed model, p-type CdTe and n-type CdS were used as an absorber layer and window layer, respectively. An additional layer of highly doped graphene [35] was applied as a back surface field (BSF) layer next to the absorber layer on the substrate, as shown in Figure 1.

The layers in this solar cell were organized according to the energy bandgap, in which the layer with a lower bandgap was placed on the bottom, while the layer with a higher bandgap was placed on the top surface [36]. The layer with a higher bandgap can absorb a wide range of solar radiation of a shorter wavelength [37]. An additional ultrathin layer of graphene as a BSF was added to reduce the recombination losses at the back contact, which enhances the efficiency of a solar cell.

### 2.2. PC1D Modeling Tool

The personal computer one-dimensional (PC1D) modeling tool is used to study the photovoltaic properties of solar cells, and it was developed by a team of the UNSW [38]. PC1D allows the simulation of the optoelectrical properties of semiconductor devices. The main advantages of PC1D include rapid calculation speeds, an intuitive user interface, and an extensive list of material and physical parameters. By varying the wavelength of the excitation light source, PC1D can calculate both current-voltage characteristics and the spectral quantum efficiency of a solar cell [39,40]. The PC1D software contains plentiful library files including numerous parameters for semiconductor devices such as GaAs, Ge, c-Si, GIN, CIGS, a-Si, AlGaAs, and InP [41,42,43]. In the simulation tool, the input key parameters, such as device area, device thickness, carrier concentration, bandgap, temperature, reflectance, etc., were used to elucidate the photovoltaic parameters of the solar cell. The detailed input parameters of this software have been summarized in Table 1. All simulations were executed under a constant light intensity of 0.1 W/cm^2^ (AM) 1.5 at 300 K temperature. For all PV simulations, the bulk recombination time was set from 1 to 100 μs and the doping concentration in the solar cell was set in the range of 1 × 10^15^ to 1 × 10^20^ cm^−3^ as reported in previous research articles [44,45,46].

## 3. Results and Discussion

### 3.1. Impact of Carrier Lifetime in BSF Layer

**Figure 2:**
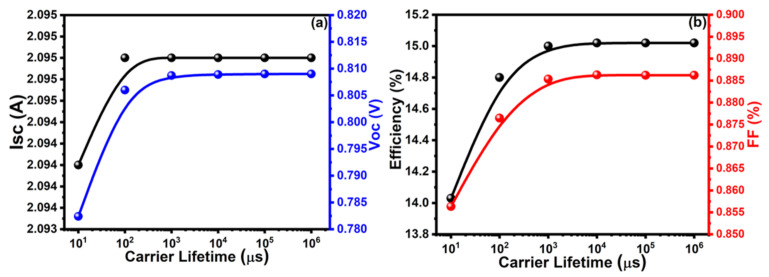
Analysis of (**a**) Isc and Voc, (**b**) efficiency and FF with carrier lifetime of BSF layer.

Carrier lifetime in the BSF layer has a very vital role in the efficiency of a solar cell. The probability of carriers reaching their respective direction’s end before their recombination is higher when the carrier lifetime is longer [50]. The photovoltaic properties, like I_sc_, V_oc_, efficiency, and FF, were characterized in the range from 1 to 10^6^ μs of carrier lifetime of the solar cells. When the carrier lifetime in the BSF layer increased from 1 to 10^3^ μs, the PV properties of solar cells increased and, after that, these factors were saturated, increasing even further. The maximum values of I_sc_ = 2.09 A, V_oc_ = 0.809 V, η = 15% and FF = 88.54% were be observed at 1 × 10^3^ μs of carrier lifetime, as shown in Figure 2a,b. Thus, the optimized value of carrier lifetime is 1 × 10^3^ μs in the BSF layer of the CdTe solar cell.

### 3.2. Impact of Doping Concentration in BSF Layer

**Figure 3:**
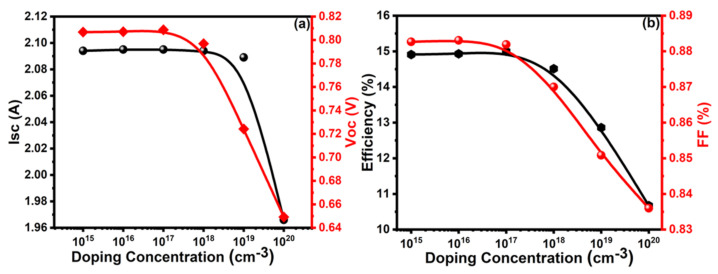
Analysis of (**a**) Isc and Voc, (**b**) efficiency and FF with a doping concentration of BSF layer.

A doping concentration is one of the decisive factors affecting the overall performance of the solar cell. The values of PV parameters such as I_sc_, V_oc_, efficiency, and FF increase with higher doping concentrations in the BSF layer due to an increase in band-bending in CdTe solar cells [51]. It is also proven that a very high doping concentration in the back contact layer can support the generation of a tunneling contact. It is known to be highly difficult to obtain high p-type doping in CdTe material due to self-compensation [52]. The PV properties (I_sc_, V_oc_, η, and FF) were characterized in the range from 1 × 10^15^ to 1 × 10^20^ cm^−3^ of doping concentration in the BSF layer of the solar cell. When the doping concentration in the BSF layer increased from 10^15^ to 10^17^ cm^−3^, the PV properties remained unchanged and, after that, started to decrease sharply with a further increase. Lower doping density leads to a wider depletion region, which is beneficial for the carrier collection and recombination process [53]. The optimum values of I_sc_ = 2.09 A, V_oc_ = 0.809 V, η = 15%, and FF = 88.55% were observed at 1 × 10^17^ cm^−3^ of doping concentration, as shown in Figure 3a,b. Thus, the optimized value of doping concentration is 1 × 10^17^ cm^−3^ in the BSF layer of the CdTe solar cell.

### 3.3. Impact of the Thickness of BSF Layer

**Figure 4:**
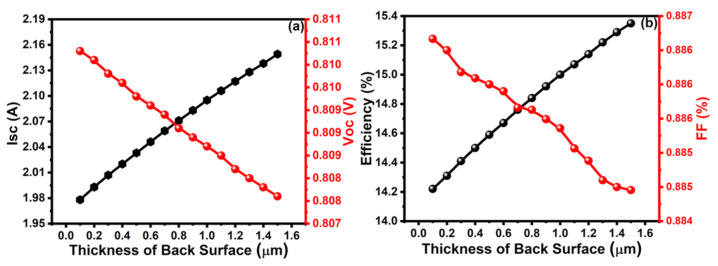
Analysis of (**a**) Isc and Voc, (**b**) efficiency and FF with the thickness of BSF layer.

It is essential to control the optimal thickness of the BSF layer since an excessive thickness can cause fast degradation due to diffusion; however, a much thinner BSF layer gives insufficient intermixing and doping in the bulk CdTe [54]. The thickness of the BSF layer should be ultrathin, which is very difficult to control. The PV properties, such as I_sc_, V_oc_, η, and FF, were characterized in the range from 0.1 to 1.5 μm of the thickness of graphene as the BSF layer of the solar cell. The value of Isc increased with an increase in the thickness of the BSF layer, whereas the value of Voc decreased, as shown in Figure 4a. Similarly, the efficiency of a cell was increased with the increase in the thickness of the BSF layer, whereas the FF value was decreased, as shown in Figure 4b, so it is difficult to set the optimum thickness of the BSF layer. For efficient and practicable solar cell fabrication, an obtainable (i.e., possible) thickness of graphene as the BSF layer should be chosen. The optimum values of I_sc_ = 2.09 A, V_oc_ = 0.808 V, η = 15%, and FF = 88.53% were observed at 1 μm thickness of the BSF layer. Therefore, the optimum value of the thickness of graphene as the BSF layer might be 1 μm for fabrication.

### 3.4. Impact of Photogeneration Rate of Carriers in BSF Layer

**Figure 5:**
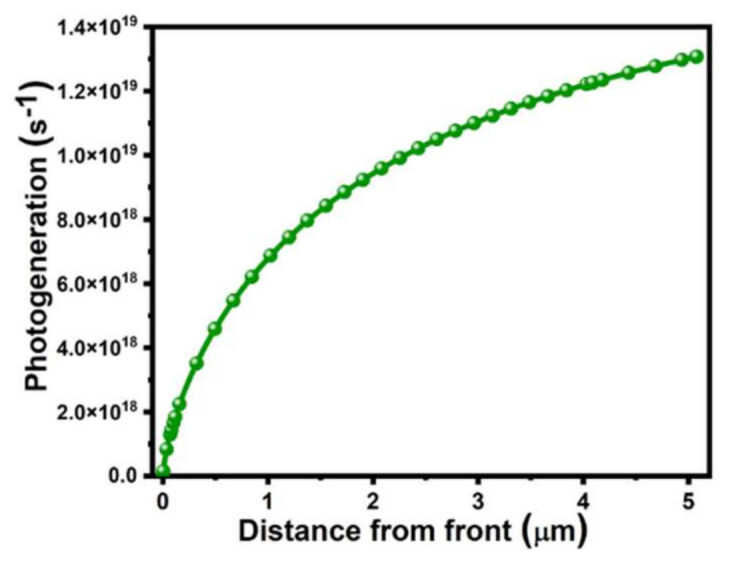
Analysis of cumulative photogeneration rate with distance from front in BSF layer.

Minority carrier transport parameters critically affect the function and performance of various p-n junction semiconductor devices with bipolar transistors and solar cells [55]. Various recombination processes were applied to find the carrier lifetime and diffusion length of the minority charge carriers in the emitter and all parts of the solar cell [56]. The thickness of the p-n junction was about 4 μm and the photogeneration rate was 1.22 × 10^19^ s^−1^ at that thickness, which is appropriate for recombination of charge carriers. The simulation result showed that the photogeneration rate increases logarithmically as the distance from the front increases, as shown in Figure 5, which might be suitable for efficient solar cells.

### 3.5. Energy Bandgap of Layers in Solar Cell

**Figure 6:**
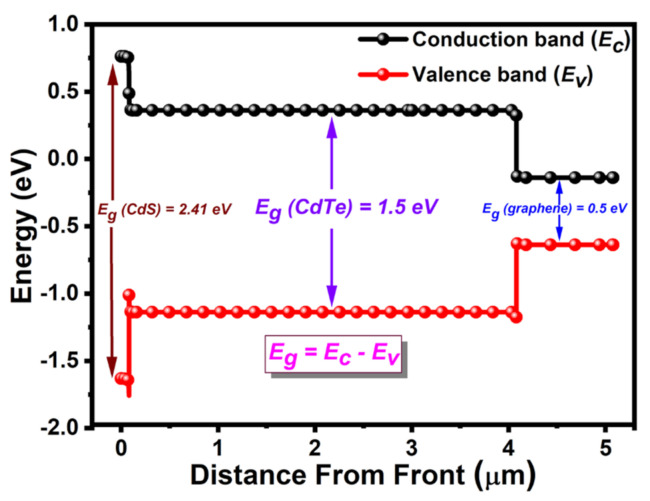
Analysis of bandgap energy of graphene BSF layer-based CdTe solar cell.

The energy bandgap is the threshold energy that is required to excite electrons up to a state in the conduction band where they can participate in conduction [57]. The theoretical values of the energy bandgap are 2.42  eV [58], 1.45  eV [59], and 0.264–0.786 eV [60] for window (CdS), absorber (CdTe), and BSF (graphene) layers, respectively, at room temperature. When light radiation with a wide range of wavelengths enters the solar cell, it must cross through different materials with various energy bandgaps. The simulated values of energy bandgaps were 2.41, 1.5, and 0.5 eV for window (CdS), absorber (CdTe), and BSF (graphene) layers, respectively, as shown in Figure 6, which almost match the theoretical values. From the simulation, it was confirmed that the proposed solar cell structure is appropriate for fabrication.

### 3.6. Photovoltaic Characteristics

**Figure 7:**
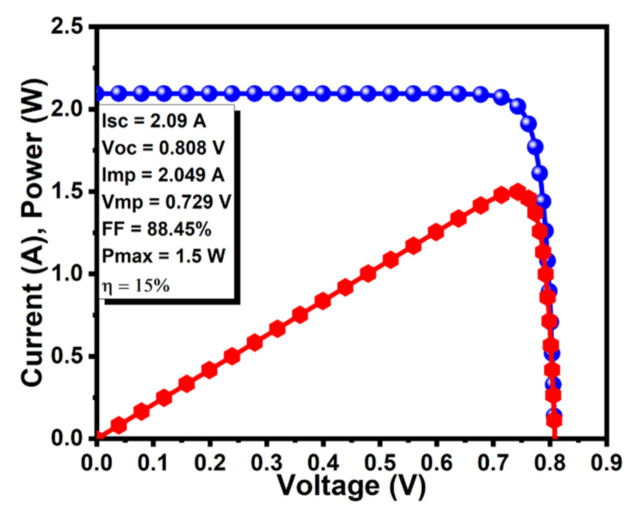
Analysis of current and power curve of graphene BSF layer-based CdTe solar cell.

**Figure 8:**
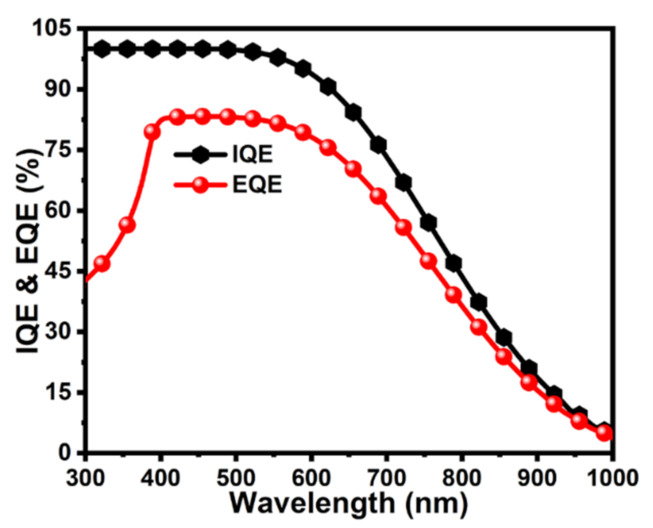
Analysis of quantum efficiency of graphene BSF layer-based CdTe solar cell.

The ultimate characteristic is the current, power, and efficiency curve to examine the overall efficiency of the solar cell. The simulation results exhibited the highest values of I_sc_ = 2.09 A, V_oc_ = 0.808 V, I_mp_ = 2.049 A, V_mp_ = 0.729 V, P_max_ = 1.5 W, and η = 15%, as shown in Figure 7. Quantum efficiency is also one of the most important characteristics to estimate the performance of the solar cell in the specific range of the wavelength. The internal quantum efficiency (IQE) was above 100% whilst an external quantum efficiency of approximately 85% was achieved in the wavelength range of 300–1000 nm, as shown in Figure 8.

The comparative study of the reported graphene back contact surface layer-based CdTe solar cells is listed in Table 2. This simulation presented impressive results and confirmed that the proposed structure of the solar cell could be suitable for efficient fabrication.

## 4. Conclusions

The possibility of ultrathin layering and high efficiency of graphene material as a back surface field (BSF)-based CdTe solar cell has been simulated successfully using the PC1D simulation tool. The highest I_sc_ = 2.09 A, η = 15% and QE~85% by CdTe solar cell were accomplished when the graphene as a BSF layer had a carrier lifetime of 1 × 10^3^ μs and doping concentration of 1 × 10^17^ cm^−3^. The obtained results suggest that the thickness of the BSF graphene layer (1 μm) is ultrathin which is an appropriate and optimal thickness for the fabrication of high-performance CdTe solar cells. Therefore, the simulation results prove that the graphene as the BSF layer with ultrathin could be highly efficient, low cost, and providing the ease of fabrication for CdTe solar cells with excellent photovoltaic properties.

## Figures and Tables

**Table 1:** Internal parameters of the PC1D simulation tool.

Parameters	CdS	CdTe	Graphene
Thickness	80 nm [7]	4 μm [7]	1 μm
Energy band gap (eV)	2.4 [47]	1.5 [47]	0.5 [48]
Electron affinity	4.2 [49]	4.28 [49]	4.7 [48]
Bulk recombination	1000 μs	1000 μs	10–10^6^ μs
Doping concentration	1 × 10^17^ cm^−3^	1 × 10^16^ cm^–3^	1 × 10^15^–1 × 10^20^ cm^−3^
Excitation mode	Transient	Transient	Transient
Constant intensity	One sun	One sun	One sun
Dielectric constant	10	9.4	7.1
Temperature	300 K	300 K	300 K
Constant intensity	0.1 W/cm^−2^	0.1 W/cm^−2^	0.1 W/cm^−2^
Primary light source	AM 1.5 D spectrum	AM 1.5 D spectrum	AM 1.5 D spectrum
Other parameters	Internal PC1D	Internal PC1D	Internal PC1D

**Table 2:** Comparative study of the performance of graphene as BSF material-based CdTe solar cell.

BSF Materials	J_sc_ [mA/cm^2^]	V_oc_ [V]	FF [%]	Efficiency [%]	References
Cu NWs/graphene	22.4	0.801	67.40	12.1	[61]
CuPs/graphene	21.3	0.805	68.10	11.7	[61]
Graphene	22.2	0.633	43.01	12.2	[62]
Graphene	40.0	0.511	65.03	13.2	[63]
Graphene	20.9	0.808	88.45	15	This work
